# Label-Free Mass Spectrometry-Based Proteomic Analysis in Lamb Tissues after Fish Oil, Carnosic Acid, and Inorganic Selenium Supplementation

**DOI:** 10.3390/ani12111428

**Published:** 2022-05-31

**Authors:** Andrzej Gawor, Anna Ruszczyńska, Anna Konopka, Grzegorz Wryk, Marian Czauderna, Ewa Bulska

**Affiliations:** 1 Biological and Chemical Research Centre, Faculty of Chemistry, University of Warsaw, Żwirki i Wigury 101, 02-089 Warsaw, Poland; agawor@chem.uw.edu.pl (A.G.); aruszcz@chem.uw.edu.pl (A.R.); a.konopka@cnbc.uw.edu.pl (A.K.); gwryk@cnbc.uw.edu.pl (G.W.); 2 The Kielanowski Institute of Animal Physiology and Nutrition, Polish Academy of Sciences, Instytucka 3, 05-110 Jabłonna, Poland; m.czauderna@ifzz.pl

**Keywords:** selenium, supplementation, label-free mass spectrometry, proteomics, protein-protein interaction

## Abstract

**Simple Summary:**

Advances in proteomics and bioinformatics analysis offer the potential to investigate nutrients’ influence on protein expression profiles, and consequently on biological processes, molecular functions, and cellular components. However, knowledge in this area, particular about the exact way selenium modulates protein expression, remains limited. Therefore, in this project, global differential proteomic experiments were carried out in order to identify changes in the expression of proteins in animal tissues obtained from lambs on a specific diet involving the addition of a combination of different supplements, namely, inorganic selenium compounds, fish oil, and carnosic acid. Following inorganic selenium supplementation, a protein-protein interaction network analysis of forty differentially-expressed proteins indicated two significant clusters.

**Abstract:**

Selenium is an essential nutrient, building twenty five identified selenoproteins in humans known to perform several important biological functions. The small amount of selenium in the earth’s crust in certain regions along with the risk of deficiency in organisms have resulted in increasingly popular dietary supplementation in animals, implemented via, e.g., inorganic selenium compounds. Even though selenium is included in selenoproteins in the form of selenocysteine, the dietary effect of selenium may result in the expression of other proteins or genes. Very little is known about the expression effects modulated by selenium. The present study aimed to examine the significance of protein expression in lamb tissues obtained after dietary supplementation with selenium (sodium selenate) and two other feed additives, fish oil and carnosic acid. Label-free mass spectrometry-based proteomic analysis was successfully applied to examine the animal tissues. Protein-protein interaction network analysis of forty differently-expressed proteins following inorganic selenium supplementation indicated two significant clusters which are involved in cell adhesion, heart development, actin filament-based movement, plasma membrane repair, and establishment of organelle localization.

## 1. Introduction

Selenium (Se) is an essential nutrient important for biological functions [1]. Selenium plays a notable role in the antioxidant defense system [2]. The various selenium compounds are known to possess anti-cancer properties [3] and have the ability to reduce the activity of viruses, including HIV [4]. Small Se amounts in the earth’s crust in certain regions [5] along with the risk its deficiency poses to living organisms has resulted in the increased popularity of dietary supplementation in animals, implemented via inorganic or organic selenium compounds [6,7,8,9,10] and even elemental selenium nanoparticles [11,12] present in food additives. The evidence for an effect of selenium supplementation on increased selenoenzymes activity exists, although any association with physiological and clinical effects is not well defined [13]. The effect of a selenium-rich diet on selenoprotein gene expression has been determined in various animals and in different tissues [14,15,16,17,18], and has been proven to depend on supplemented forms of Se [16]; investigation of chicken tissues has demonstrated that 2-hydroxy-4-methylselenobutanoic acid takes part in the regulation of selenoprotein gene expression (SELENOP, SELENOU and GPX4) when compared to other selenium forms, such as sodium selenate and selenised yeast. Several studies have confirmed that Se may predominantly affect the genes encoding proteins and plays a role in protein biosynthesis, e.g., increased Se consumption caused the growth of selenoproteins production and strengthened lymphocyte function, which is related to the upregulation of ribosomal protein and translation factor genes [19]. Even though Se is incorporated into selenoproteins as selenocysteine during translation, the dietary effects of selenium supplementation may result in the expression of other proteins or genes. Although knowledge on the modulation of expression as a result of Se nutrition remains unexplained, advanced genomic technology provides the opportunity to examine gene expression profiles and their influence on cellular function. Therefore, in order to identify changes in protein expression, the evaluation of the results of quantitative protein analysis is carried out here using powerful bioinformatics software tools [20,21,22].

Naturally low selenium concentrations in biological samples as well as their complex matrix make speciation analysis challenging, especially when quantitative analysis is considered. The determination of the total content of trace amounts of Se, which is essential in respect of the extraction efficiency, is not problematic if a sufficiently sensitive and selective detection method is used [23,24,25,26,27,28]. When thinking about speciation, one must consider the combination of an effective separation followed by well-designed detection. First, high-pressure liquid chromatography (HPLC) seems to be most suitable, especially when combined with ICP-MS (HPLC-ICP-MS) [15,29,30]. In the case of HPLC-ICP-MS, the compatibility of the retention times of respective standards with those of the compounds used is considered to be proof of its presence in a sample. Although this is admittedly sufficient evidence of the presence of a particular substance for which standards are available, many signals remain unspecified due to lack of availability of the corresponding standards [30]. Thus, HPLC-ICP-MS as such is not suitable for proteomic analysis and needs to be verified by additional studies in order to avoid mistakes resulting from overlapping signals from different compounds occurring at the same retention time. Essential confirmation is possible with the use of tandem mass spectrometry (MS/MS) equipped with an electrospray ionization source (ESI) [30].

Mass spectrometry-based (MS) proteomics has grown significantly within the last several years. The preparation of samples, the efficiency of separation techniques, and the continuously evolving and improving performance of instruments for accurate high-resolution mass analysis (HRMA) play an important role in proteomic analysis. Furthermore, newly developed computational algorithms as along with progressively improving development and validation of databases have enhanced the effectiveness and reliability of protein identification. Recent rapidly-developing innovative labelling [31] and label-free [14,32,33,34] techniques and appropriate proteomic software have strengthened the accuracy of quantitative analysis. In fact, such development and expansion have provided the opportunity to identify more up- or downregulated proteins, e.g., in experiments exploring the effects of different supplemental components, thereby advancing a deeper comprehension of biological processes. Considering the extensive amount of data generated from a single proteomic analysis, it is crucial to use specific algorithms in order to identify expression patterns that correlate with a particular biological/pathological phenotype from multiple samples [35]. The methodology described above can be used to explain how different forms of nutrients moderate their impact on tissue Se content and gene expression in animals. Moreover, advanced technologies can be used both to indicate changes in protein expression as a result of diet [36] and to identify specific biomarkers related to meat production [37,38]. Proteomic or metabolomic assays can additionally be used to monitor the health of farm animals, such as horses [39,40,41], greyhounds [42,43], camels [44] and chicken [38].

The objective of the project was to utilize advanced bioinformatics tools in order to evaluate results obtained via label-free proteomics analysis of heart tissues retrieved from lambs fed a diet enriched with an inorganic selenium compound, sodium selenate (Se(VI)), with the addition of fish oil (FO) and carnosic acid (CA). The additives in the lambs’ diets did not adversely affect the animal’s well-being or overall state of health, as no pathological or macroscopic variation was observed in the examined lambs in our previous studies [7,8,9,15]. According to the current literature, no previous studies have examined the effects of a combination of FO and CA with selenium compound supplementation on global changes in protein expression. Considering the few publications with results in farm animal samples, a continuation of the earlier studies [14,15] was proposed.

## 2. Materials and Methods

### 2.1. Sheep, Rations, Dietary Supplementation, and Tissue Collection

All nutritional experiments were carried out on male sheep (Corriedale) fed with rations including extra Se supplementation. All investigations were conducted on sheep in accordance with the guidelines of the third Local Commission of Animal Experiment Ethics located at the Warsaw University of Life Sciences (WULS), Poland. Welfare guidelines and animal handling dealing were carefully adhered to throughout the whole period of our investigations conducted on animals. All nutritional studies on sheep and heart collections were performed in professional farm animal laboratory rooms located at the Kielanowski Institute of Animal Physiology and Nutrition (Polish Academy of Sciences) in Jabłonna near Warsaw (Poland) [14,16,37].

Thirty sheep in the age range of 82–90 days (average body weight of sheep: 24.3 kg ± 1.6 kg) at the start of studies were individually located in pens; the length, width, and height of each pen were 170 cm, 130 cm, and 150 cm, respectively. During a three-week initial period, all sheep had free and unlimited access to drinking water (tap water) as well as to a basal diet (BD) supplemented with a mixture containing vitamins and minerals (20 g/kg of the BD), rapeseed oil (30 g/kg of the BD or 20 g/kg of the BD), and odourless FO (10 g/kg of the BD) [15].

The BD is the standard ration (a concentrated hay ration) consisting of the following components: meadow hay, a mixture of barley meal and soybean meal, wheat starch, and a mixture containing vitamins and minerals (aPL–1 405 002 p). The chemical composition of the ingredients in the BD and the fatty acid levels in rapeseed oil (RO) and odourless FO have been presented in previous papers [15].

Individuals (six per each experimental group) from the following groups were randomly selected for the study: Group 0 (20 g of RO, 10 g of FO and 1 g of CA in 1 kg of the BD) was the control group and Group SeVI (20 g of RO, 10 of FO, 1 g of CA and 0.35 mg Se as Se(VI) in 1 kg of the BD) was named the inorganic selenium supplementation group. Importantly, Se(VI) is a less reactive compound than selenite. In fact, dietary selenite can react in the digestive tract, particularly in a rumen, with dietary components, especially those with thiol groups or disulfide groups like cystine [9,45,46]. The product of these reactions is elemental Se (Se^0^), which in the anaerobic ruminal environment is unreactive. As a consequence, Se^0^ is efficiently excreted in faeces of lambs.

After a 35-day period of feeding with the experimental diets followed by fasting for 12 h, all sheep were rendered unconscious via xylazine (i.e., intra-muscular injections: ~ 0.4 mg xylazine per kg of lambs’ body mass). Next, animals were rapidly slaughtered. Anaesthesia of the sheep and collection of selected tissues were performed in accordance with the Regulation of the European Union Council (No. 130 1099/2009; 24 September 2009) on the Protection of Animals at the Time of Killing and in accordance with the third Local Commission of Animal Experiment Ethics located at WULS (Warsaw, Poland; protocol code: 41/2013; date of approval: 17 July 2013). After the sheep were euthanized, heart tissues were removed immediately from each animal along with the internal organs and then each organ was homogenized. All tissue samples for proteomics analysis were placed into tightly closed containers. These containers were immediately frozen to −80 °C to await analytical investigation. Only heart samples were used for these experiments. The total content of selenium in heart tissues in Group 0 and in Group SeVI was, respectively, (871.2 ± 59.5) µg/kg and (1138.5 ± 103.5) µg/kg, as previously published [15]. The details concerning the chemical profiles of the heart tissues from both groups have been well-described in previously published studies [15].

### 2.2. Dietary Supplements, Reagents, and Analytical Tools

Analytical-grade reagents, chromatographic solvents, and standards were obtained from Promega (Madison, WI, USA), Merck (Darmstadt, Germany), Thermo Scientific (Bartlesville, OK, USA), and EMD Millipore (Madison, WI, Germany). Deionized water obtained from the Milli-Q system (18.2 MΩ cm; EMD Millipore; Darmstadt Germany) was used for samples and standard dilution. The dietary supplement carnosic acid (CA) was supplied by Hunan Geneham Biomedical Technology Ltd. (The People’s Republic of China; Changsha Road), and odourless FO (enriched in n-3LPUFA) and RO were obtained from the company “AGSOL” (Pacanów, Poland). The energy levels of RO and odourless FO were 37.02 MJ/kg of RO and 36.81 MJ/kg of FO. Samples of the vitamin and mineral premix were purchased from POLFAMIX OK by Trouw-Nutrition (Grodzisk-Mazowiecki, Poland).

The analytical instrumentation used for sample preparation was as follows: an Ultra-Turrax mechanical homogenizer (IKA, Königswinter, Germany), CLN 240 laboratory incubator (MultiSerw, Brzeźnica, Poland), SpeedVac Concentrator Plus vacuum concentrator (Eppendorf, Enfield, CT, USA), 5804/5804 R centrifuge (Eppendorf, Enfield, CT, USA), vortex shaker (IKA, Königswinter, Germany), and Eppendorf Comfort thermomixer (Eppendorf, Enfield, CT, USA). An in-house-packed capillary C-18 column (75 μm × 500 mm, particle size 1.9 μm) (Dr. Maisch, Ammerbuch, Germany) was employed for peptide separations using a nano-UHPLC system (Dionex Ultimate 3000 RSLC, Thermo Scientific, Enfield, CT, USA) coupled to a high-resolution tandem mass spectrometer (Orbitrap Fusion Tribrid™ Mass Spectrometer, Thermo Scientific, Enfield, CT, USA).

### 2.3. Sample Preparation, LC-MS/MS Analysis, and Data Analysis for Relative Protein Quantification

Sample preparation for proteomics study and the conditions of liquid chromatography with tandem mass spectrometry (LC-MS/MS) were described in detail in our recent paper [34]. Analysis of MS/MS raw data was performed using MaxQuant version 1.6.1.0 (Max-Planck-Institute of Biochemistry, Martinsried, Germany) and searched by the Andromeda search engine [47,48]. The *Ovis aries* sequence was obtained from the UniProt database (n = 23,111 protein isoforms, retrieved December 2021). Fixed modification, which derived from acrylamide as an alkylating agent, was as follows: for propionamidation on cysteine, Se-cysteine, Se-methionine, and oxidation of Se-cysteine, Se-methionine and oxidation of methionine and acetylation of protein N-terminal were set as variable modifications. Trypsin was set as a site-specific enzyme with no more than two missed cleavage sites. In the main search, mass tolerances of 5 ppm for parent ions and 10 ppm for fragment ions were acceptable. As an instrument type, an Orbitrap was selected. Due to the fact that labelling was not carried out, the multiplicity was set at one. Only peptides at least seven amino acids in length were used for evaluation. The search for common contaminants is considered in the operating protocol of the MaxQuant algorithm. The IDs were each filtered at a false discovery rate (FDR) of 0.01 at the PSM (peptide spectrum matching) level as well as at the protein level, using a target–decoy method to search for false discoveries. The quantification of proteins was assessed using label-free quantification (LFQ), including only razor and unique peptides. In this case, LFQ was chosen, with MS/MS as required for LFQ matching. Concerning protein quantification, a minimum ratio count of two was set. However, the other settings of MaxQuant were configured as their defaults, following the protocol proposed by Tyanova et al. [48]. The intensity of LFQ is the relative quantification of the protein within all samples, and is used instead being represented by the normalised intensity profile generated following the MaxQuant algorithms [49].

Any files retrieved using MaxQuant were subsequently analysed using the Perseus framework, available online at http://www.perseus-framework.org [50], accessed on 31 December 2021. Perseus software (version 1.6.14.0, Max-Planck-Institute of Biochemistry, Martinsried, Germany) was utilised to complete the bioinformatics and statistical analysis, employing the output files from MaxQuant. All MaxQuant data were further filtered for protein identifications based on sites only, potential contaminants, and reverse identifications (false positives). Converted LFQ intensities were then transformed into logarithmic values, and missing data were overwritten by the imputation of missing values following a normal distribution. An additional stage was the conversion of the LFQ intensity ratio into log2. After that, the rows were filtered against valid values, with at least eight values in at least one group. Any missing values were replaced by a normal distribution using the imputation feature. Finally, the mean LFQ intensity and standard error of the mean were computed for all experimental groups. In order to achieve an estimation of the variabilities between biological replicates of the examined tissue samples, Student’s *t*-test was implemented using Perseus software with very stringent statistical criteria: FDR = 1%; s0 = 1; adjusted *p* < 0.01.

### 2.4. Metrological Aspects of Relative Protein Quantification

A reproducible chromatographic separation is the most essential requirement in reliably comparing different runs, and provides sufficient quantitative information concerning the analysed proteome. Prior to analysis, it is important to check and examine the working conditions for the chromatography and the stability of the selected parameters, such as peak widths, peak shapes, resolution, and retention time. The LC-MS/MS instrument was verified on a daily basis while performing accurate mass measurements of peptides and proteins. In order to check the stability of mass spectrometry during measuring sequences, a quality control sample (QC) was prepared by mixing all the samples from the whole experiment. The use of complex control solutions increases the probability of peptide co-elution, which impedes the process of data analysis. The QC sample was measured before and after experiments and between each experimental group.

## 3. Results

**Figure 1:**
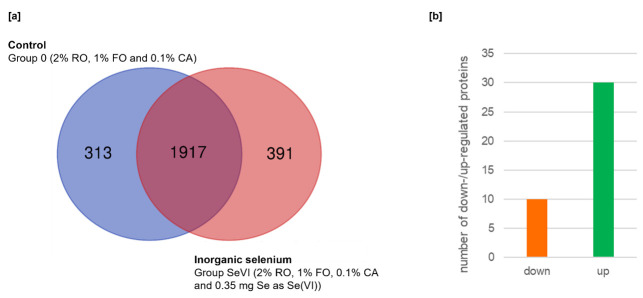
Overview of proteomics analysis: (**a**) the overlap between all identified proteins in Group 0 and Group SeVI, shown as a Venn diagram; (**b**) The number of up- and downregulated proteins obtained by comparing Group 0 and Group SeVI.

In the course of LC–MS/MS analysis in the present study, 2230 proteins (13764 unique peptides) and 2308 proteins (13858 unique peptides) were detected in Group 0 and Group SeVI, respectively. The overview of the proteomic analysis shown in Figure 1 indicates less than 20% characteristic for each group of proteins among all identified. There were 1917 proteins common to groups 0 and SeVI, while 313 proteins (14%) were unique to Group 0 and 391 (17%) to Group SeVI (Figure 1a). Through data analysis, a significant difference in the expression level of forty proteins was found between the examined groups (Figure 1b; Table 1) after applying very stringent statistical criteria (FDR = 1%; s0 = 1; adjusted *p* < 0.01).

**Figure 2:**
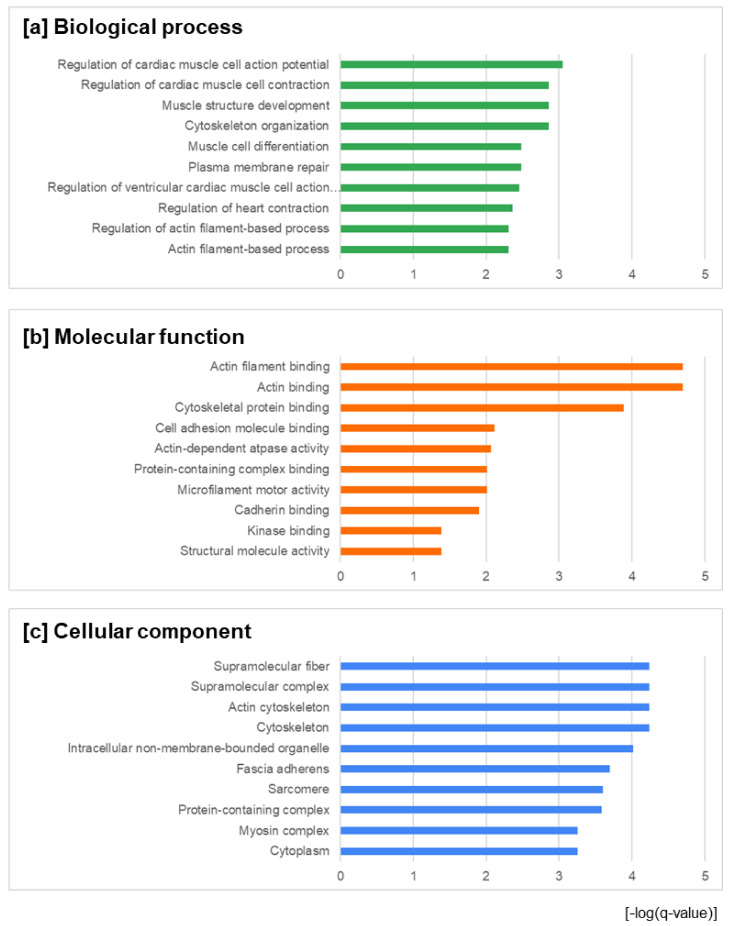
Gene ontology enrichment analysis of the identified differentially-expressed proteins in three categories: (**a**) biological process; (**b**) molecular function; and (**c**) cellular components. The abscissa was the –log (q-value) in order to show the significance between the proteins involved in feature function and annotation; this parameter describes the significance of the enrichment. The displayed q-values are corrected FDR values for multiple testing within each category, for which the Benjamini–Hochberg procedure was used.

Following this step, a gene ontology (GO) enrichment analysis was applied to the identified differentially-expressed proteins. Gene ontology analysis classifies function into the categories of cellular components, molecular function, and biological process. The top ten enrichments in (i) the biological processes category were regulation of cardiac muscle cell action potential, cytoskeleton organization, muscle structure development, regulation of cardiac muscle cell contraction, plasma membrane repair, muscle cell differentiation, regulation of ventricular cardiac muscle cell action potential, regulation of heart contraction, actin filament-based process, and regulation of actin filament-based process (Figure 2a); in (ii) the molecular function category they were actin filament binding, actin binding, cytoskeletal protein binding, cell adhesion molecule binding, actin-dependent ATPase activity, microfilament motor activity, protein-containing complex binding, cadherin binding, structural molecule activity, and kinase binding (Figure 2b); and in (iii) the cellular component category they were cytoskeleton, actin cytoskeleton, supramolecular complex, supramolecular fiber, intracellular non-membrane-bounded organelle, fascia adherens, sarcomere, protein-containing complex, cytoplasm, and myosin complex (Figure 2c). A GO classification was performed for all identified high-confidence differentially-expressed proteins using the STRING environment (http://string-db.org/), accessed on 31 December 2021.

**Figure 3:**
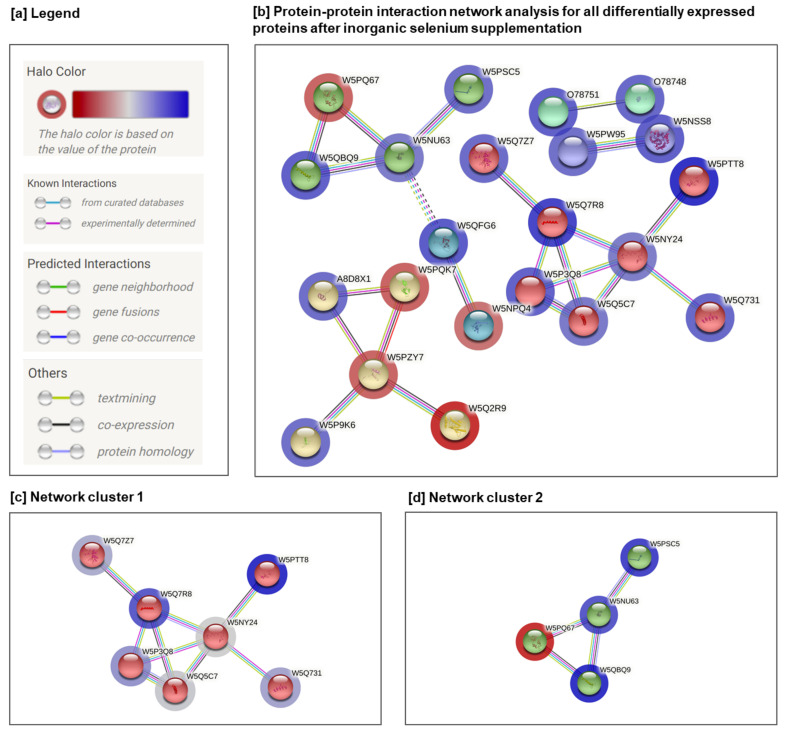
Protein-protein interaction network of differentially regulated proteins after inorganic selenium supplementation with the addition of 2% RO, 1% FO, 0.1% CA: (**a**) legend; (**b**) protein–protein interaction network analysis for all differentially expressed proteins; (**c**,**d**) two interactions between protein complexes from the STRING database formed by means of the Markov Cluster Algorithm. The number of lines represents the strength of predicted functional interactions between proteins.

Protein-protein interaction (PPI) network analysis supports an understanding of the biological responses of inorganic selenium supplementation. The STRING framework [22] was employed to establish comprehensive networks using the same criteria as applied in the GO analysis. Regarding protein interactions, the minimum required interaction score was assigned a high confidence of 0.7, and the maximum additional interactors were determined to be 0. Abbreviated protein names used in the networks are listed in Table 1. The protein-protein interaction results obtained from the STRING environment illustrate the association of differentially-expressed proteins, with other major associations shown in Figure 3.

The PPI analysis demonstrated 40 nodes (proteins) and 157 edges, with the following statistical parameters: (i) average node degree, 2.7; (ii) average local clustering coefficient, 0.494; (iii) expected number of edges, 28; and (iv) PPI enrichment *p*-value, 1.29 × 10^−5^ Essentially, this means that proteins have significantly more interactions among themselves than was expected from a random set of proteins of the same size and degree distribution drawn from the genome. However, such enrichment highlights that these proteins are at least in part biologically associated as a group. The identified clusters are shown coloured in red, green, and yellow. The solid and dotted lines indicate connections within the same and different clusters, respectively. Different colours indicate different types of interaction: cyan were from curated databases; pink were experimentally determined; blue from gene co-occurrence; red from gene fusion; green from gene neighbourhood; light blue from protein homology; black from co-expression; and yellow from text mining. The level of expression is depicted with a coloured halo of proteins (Figure 3a,b).

The cluster analysis of forty differentially-expressed proteins indicated several significant clusters found with the Markov Cluster Algorithm (MCL) [51]. Among these, network cluster 1 (Figure 3c) contained seven proteins (average local clustering coefficient: 0.829; PPI enrichment *p*-value: 3.8 × 10^−10^) network cluster 2 (Figure 3d) contained four proteins (average local clustering coefficient: 0.833; PPI enrichment *p*-value: 8.07 × 10^−9^), respectively. Other identified clusters (yellow, blue, and violet) either consisted of a small number of proteins or no significant enrichment was detected between interactions.

## 4. Discussion

The function analysis of cluster 1 indicated the following group of proteins: junction plakoglobin (W5Q7R8); four and a half LIM domains 2 (W5PTT8); and desmoplakin (W5Q7Z7). In the GO analysis of the biological processes, cluster 1 was mostly enriched in cell adhesion (FDR = 0.00026); bundle of cell purkinje myocyte adhesion, involved in cell communication (FDR = 0.0074); and heart development (FDR = 0.0083). The abundance of these proteins respectively increased by ↑+2.11, ↑+2.43, ↑+1.50 when comparing the control group with the inorganic selenium supplementation group. The four and a half LIM domains 2 (W5PTT8) protein belongs to a large family of LIM domain-containing proteins that are involved in a broad spectrum of functions, including cell identity, differentiation, and growth control [52]. The LIM domain is a cysteine-rich zinc-binding motif that contains a double zinc finger domain (C2CH and C4). Reflecting this dual nature of the FHL2 is that it can act as a repressor or activator of transcriptional activity depending on the cell- type [53]. This functional diversity of the FHL2 is a result of its structural configuration as an LIM-only protein. The LIM domains are non-enzymatically active in protein–protein interaction as well as being crucial for the function of LIM proteins as adaptor molecules or scaffold proteins. As a result of the selective utilisation of different LIM-domains for protein–protein interactions, FHL2 can interact with a wide range of functionally unrelated proteins, activating various signalling pathways [52].

Another key protein in cluster 1 is junction plakoglobin (W5Q7R8), which is a common junctional plaque protein. The membrane-associated plaques are strategic structural elements that affect the distribution and function of either the cytoskeleton or the cells [54]. However, the presence of plakoglobin in the desmosomes as well as in the intermediate junctions strongly suggests a major role of plakoglobin in the structure and function of submembrane plaques. A plakoglobin acts as a substrate for the vascular endothelial protein tyrosine phosphatase (VE-PTP), and is needed to promote VE-cadherin function in endothelial cells. The plakoglobin can substitute beta-catenin in E-cadherin/catenin adhesion complexes, considered to be a conjugating factor between cadherins and the actin cytoskeleton. The last protein of interest in network cluster 1 is desmoplakin DSP (W5Q7Z7), which is encoded by the DSP gene. It is a major desmosome component that is abundant and richly presented in myocardial tissue. As such, three isoforms of DSP exist, which perform a functional role in contributing to structural stability via intercellular adhesion [55]. In addition, desmoplakin has been reported [55] to regulate the transcription of adipogenic and fibrogenic genes and to maintain proper electrical conductivity through the regulation gap junctions and ion channels. A desmoplakin is essential for normal myocardial development and the maintenance of its structural functions [56]. The increased abundance of the mentioned proteins in cluster 1 in the control group in comparison with inorganic selenium supplementation suggests that the process of cell adhesion and maintenance of structural functions in the heart become less efficient after inorganic selenium supplementation.

Interestingly, the myosin family proteins myosin light chain 4 (W5PQ67; ↑↓−1.51), myosin heavy chain 10 (W5NU63; ↑+1.26), myosin IC (W5PSC5; ↑+1.46), and myosin heavy chain 9 (W5QBQ9; ↑+1.69), which have close interactions, were found in the network as cluster 2 (Figure 3d) in the GO analysis, indicating actin filament-based movement (FDR = 0.0017), plasma membrane repair (FDR = 0.0117), and establishment of organelle localization (FDR = 0.0470). All of the mentioned proteins belong to the myosin superfamily, which is a large and diverse protein family involved in several cellular pathways [57,58]. Myosin family proteins are mostly associated with membranes [58]; thus, they play additional roles as motors, especially for the transport of membranous organelles within actin filaments. Transport of vesicles to their destinations is executed by motor proteins, which move along microtubules or microfilaments. One of the superfamilies of such motor proteins are the myosins [59]. While kinesins and dyneins transport vesicles along microtubules, myosins carry out the transport along microfilaments. Nedvetsky et al. [59] have reported that the myosin Vb plays a crucial role in the aquaporin-2 recycling cycle. Aquaporins are transmembrane channel proteins, which facilitate the passive and bidirectional diffusion of water and/or small and noncharged compounds across biological membranes [60], including the selenium compounds [61] that may be associated in this study with a selenium-containing diet.

However, the expression of myosin chain family proteins was found to either increase or decrease depending on the type of proteins, which is in contrast to the results reported in the literature [62]. Fernández-Lázaro et al. [62] found that no significant effects were observed in myosin heavy chain expression in muscle tissue after oral selenium supplementation with 180 µg/day or 240 µg/day in the form of selenomethionine or with 200 µg/day in the form of sodium selenite, although the changes in protein expression observed in their study might have been different if the analysis was done on heart tissues only, as in the present study. Gene ontology enrichment analysis performed for the identified differentially-expressed proteins (Figure 2) indicated biological processes including plasma membrane repair, muscle structure development, and cytoskeleton organization, which confirms the positive effects of a diet enriched in Se on body function reported in several previous studies [15,63,64,65,66,67,68,69,70,71]. The cytoskeleton has been indicated as a biological process in functional annotation analysis of differentially expressed genes as a result of high dietary selenium supplementation in sheep reported by Elgendy et al. [72].as.

It is worth emphasizing that similar proteomic studies of the effects of selenium supplementation have been conducted on humans, although these were limited to plasma samples. Sinha et al. [73] reported that in healthy male subjects, 22 proteins were significantly altered following Se-Yeast supplementation compared to 13 proteins that were significantly changed after placebo-yeast supplementation. In their study, the differentially expressed proteins were involved in complementary and coagulation pathways, immune functions, lipid metabolism, and insulin resistance.

In general, the above-mentioned studies demonstrate that Se nutrition has a significant effect on protein expression in animals. The results of the conducted studies indicate the of changes in protein expression under the action of diets enriched in selenium in addition to other supplements. The performed proteomic analysis and protein–protein interactions may be useful in exploring the effects of this type of supplementation on the body for further studies in this field.

## 5. Conclusions

A label-free mass spectrometry-based proteomic approach was performed for the identification of several differentially-expressed proteins following inorganic selenium supplementation. An application of protein–protein interaction network analysis on forty differentially-expressed proteins indicated two significant clusters involved in cell adhesion, heart development, actin filament-based movement, plasma membrane repair, and establishment of organelle localization. A number of biological processes in which the identified proteins are involved were confirmed in the relevant literature. Furthermore, the elevated expression of proteins was found to be connected in two significant clusters involved in cell adhesion, heart development, actin filament-based movement, plasma membrane repair, and establishment of organelle localization.

## Figures and Tables

**Table 1:** The list of up- (↑) and down- (↓) regulated proteins in Group 0 (2% RO, 1% FO and 0.1% CA) in comparison with Group SeVI (2% RO, 1% FO, 0.1% CA and 0.35 mg Se as Se(VI) in 1 kg BD); FDR = 1%; s0 = 1; adjusted *p* < 0.01.

Difference	Protein IDs	Protein Names	-Log *p*-Value	Seq. Coverage [%]	Mol. Weight [kDa]
↑+2.43	W5PTT8	Four and a half LIM domains 2	1.86	45	32.859
↑+2.11	W5Q7R8	Junction plakoglobin	3.80	27	81.961
↑+1.76	W5QFG6	Actin related protein 2	4.16	19	52.197
↑+1.71	W5NTG3	Glycine cleavage system H protein	2.81	27	19.109
↑+1.70	W5NSS8	Tubulin beta chain	3.69	61	47.985
↑+1.69	W5QBQ9	Myosin heavy chain 9	4.08	33	220.830
↑+1.68	O78751	ATP synthase protein 8 (A6L) (F-ATPase subunit 8)	1.71	55	7.910
↑+1.68	W5P3Q8	Catenin alpha 3	4.00	33	63.223
↑+1.65	W5PK85	EMAP like 2	3.98	17	68.515
↑+1.56	W5Q731	59 kDa serine/threonine-protein kinase (Beta-integrin-linked kinase)	5.13	30	51.421
↑+1.56	O78748	NADH-ubiquinone oxidoreductase chain 2 (EC 7.1.1.2)	2.52	15	39.128
↑+1.53	W5P0Y1	Ryanodine receptor 2	4.35	27	544.710
↑+1.50	W5Q7Z7	Desmoplakin	2.83	35	317.880
↑+1.48	A8D8X1	60S ribosomal protein L10 (Protein QM homolog)	2.15	31	24.603
↑+1.47	W5NY53	Mannose-6-phosphate isomerase (EC 5.3.1.8)	2.68	34	55.469
↑+1.46	W5PSC5	Myosin IC	3.64	25	121.400
↑+1.45	W5PW95	Tubulin alpha chain	3.85	39	54.886
↑+1.45	W5QHK3	Methylcrotonoyl-CoA carboxylase 1	3.79	29	77.352
↑+1.44	W5PQG5	Adenylyl cyclase-associated protein	2.98	38	53.957
↑+1.44	W5P9K6	Proteasome 26S subunit, ATPase 3	2.12	29	50.645
↑+1.37	W5PPG2	Dysferlin	2.41	24	234.250
↑+1.30	W5Q5C7	Catenin alpha 1	2.45	27	100.090
↑+1.30	W5NZ80	Niban apoptosis regulator 1	2.63	17	103.360
↑+1.27	W5Q7M7	Heterogeneous nuclear ribonucleoprotein A3	3.91	37	39.709
↑+1.26	W5NU63	Myosin heavy chain 10	3.24	27	229.100
↑+1.25	W5QCD6	Isocitrate dehydrogenase [NADP] (EC 1.1.1.42)	2.95	32	46.733
↑+1.25	W5NY24	Catenin beta 1	5.08	33	85.609
↑+1.23	W5P5A0	Filamin A	4.26	24	279.600
↑+1.21	W5QGW8	Phosphodiesterase (EC 3.1.4.)	2.98	22	92.055
↑+1.15	W5PUT6	Clathrin heavy chain	3.84	32	191.880
↓−1.32	W5NPQ4	F-actin-capping protein subunit alpha	9.33	64	36.331
↓−1.38	W5Q8I4	ST13 Hsp70 interacting protein	2.05	20	43.341
↓−1.51	W5PQ67	Myosin light chain 4	1.55	72	21.296
↓−1.51	W5PZY7	Ubiquitin carboxyl extension protein 80	1.99	39	19.543
↓−1.52	W5P7E8	Dual specificity protein phosphatase (EC 3.1.3.16)	3.12	24	18.843
↓−1.54	W5PQK7	Eukaryotic translation initiation factor 5A (eIF-5A)	1.72	43	17.171
↓−1.88	W5Q4D9	NAD(P)(+)--arginine ADP-ribosyltransferase (EC 2.4.2.31)	2.56	19	43.748
↓−1.89	W5Q5Z9	MICOS complex subunit	3.11	18	23.119
↓−1.92	W5NUL7	AFG3 like matrix AAA peptidase subunit 2	2.95	20	89.411
↓−2.28	W5Q2R9	UBC core domain-containing protein	4.68	61	17.394

## Data Availability

The authors confirm that the data supporting the findings of this study are available within the article. Raw data that support the findings of this study are available from the first author upon reasonable request.
